# *Drosophila*: An Emergent Model for Delineating Interactions between the Circadian Clock and Drugs of Abuse

**DOI:** 10.1155/2017/4723836

**Published:** 2017-12-17

**Authors:** Aliza K. De Nobrega, Lisa C. Lyons

**Affiliations:** Department of Biological Science, Program in Neuroscience, Florida State University, Tallahassee, FL 32306, USA

## Abstract

Endogenous circadian oscillators orchestrate rhythms at the cellular, physiological, and behavioral levels across species to coordinate activity, for example, sleep/wake cycles, metabolism, and learning and memory, with predictable environmental cycles. The 21st century has seen a dramatic rise in the incidence of circadian and sleep disorders with globalization, technological advances, and the use of personal electronics. The circadian clock modulates alcohol- and drug-induced behaviors with circadian misalignment contributing to increased substance use and abuse. Invertebrate models, such as *Drosophila melanogaster*, have proven invaluable for the identification of genetic and molecular mechanisms underlying highly conserved processes including the circadian clock, drug tolerance, and reward systems. In this review, we highlight the contributions of *Drosophila* as a model system for understanding the bidirectional interactions between the circadian system and the drugs of abuse, alcohol and cocaine, and illustrate the highly conserved nature of these interactions between *Drosophila* and mammalian systems. Research in *Drosophila* provides mechanistic insights into the corresponding behaviors in higher organisms and can be used as a guide for targeted inquiries in mammals.

## 1. Introduction

### 1.1. Alcohol and Drug Abuse

The long-term chronic abuse of alcohol and other drugs has adverse consequences for individual health, society, and the economy [1–3]. Alcohol is one of the most commonly used and abused drugs in the United States [4] and the world [5]. As of 2014, 17 million Americans have an alcohol use disorder (AUD) representing 79% of the people diagnosed with substance use disorders, and additional 2.6 million (12.1%) have comorbid AUD and illicit drug use disorder [4]. Alcohol and other drugs of abuse collectively account for ~75,000 deaths annually in the US [6, 7]. In the United States, the health and economic costs associated with alcohol abuse are estimated at approximately $223 billion annually [1] with costs associated with other drugs of abuse including tobacco, illicit drugs, and prescription opioids collectively estimated at approximately $571.6 billion annually [8–10]. In the past few years, cocaine use has reemerged as a public health problem with a 26% increase in the number of new users in 2015 compared to 2014, with the greatest increase in users occurring among young adults [11]. Understanding the factors that contribute to alcohol and substance abuse and addiction and drug pathologies is critical for the development of therapies for the prevention and treatment of substance abuse disorders.

### 1.2. The Link between the Circadian Clock and Drug Use

**Figure 1:**
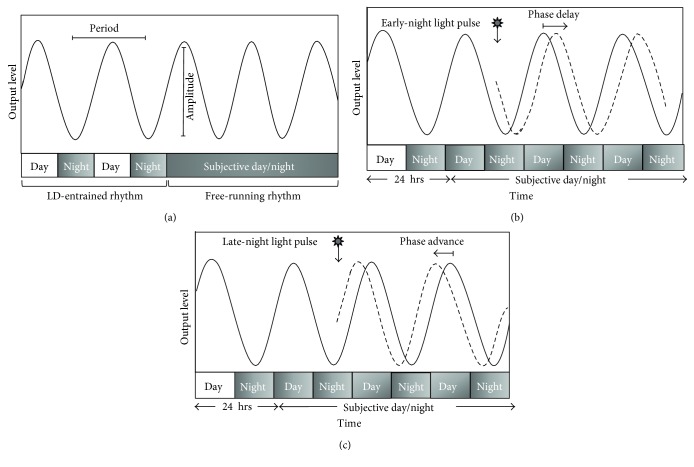
Measures of the circadian rhythm. (a) Cycles of peaks and troughs of activity occur at approximately 24-hour intervals. The period of the cycle is the time between successive peaks (or troughs) of activity whereas the extent of the increase or decrease in activity represents the amplitude of the cycle. (b) An early-night light pulse results in a phase delay. (c) A late-night light pulse results in a phase advance.

**Figure 2:**
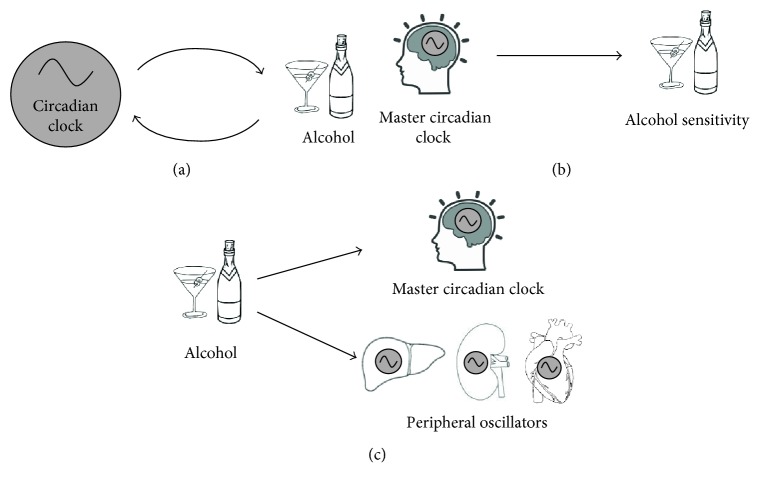
The bidirectional relationship between the circadian clock and alcohol. (a) The circadian clock modulates alcohol sensitivity and alcohol consumption. Alcohol acts upon circadian oscillators to affect phase shifting of oscillators as well as expression patterns of circadian genes leading to circadian dysfunction. (b) The master circadian clock in the brain modulates the behavioral sensitivity to alcohol including hyperactivity, sedation, recovery, and tolerance. (c) Alcohol affects the master circadian clock in the SCN as well as in peripheral oscillators in the liver, kidney, and heart.

From bacteria to humans, circadian clocks regulate cellular, physiological, and behavioral rhythms in coordination with the natural light-dark cycle (Figure 1). In addition to light, entrainment of the peripheral circadian system can be mediated by food intake schedules, exercise, or social activity [12–14]. The circadian clock modulates rhythms in metabolism, gene expression, hormone production, cell regeneration, and brain wave activity [15–17]. In the past two decades, the importance of the circadian clock in modulating alcohol and drug use and the associated pathologies has become more apparent (Figure 2). Individuals with an evening chronotype, the behavioral pattern reflecting an individual's circadian phase, exhibit higher levels of alcohol use [18, 19] and increased drug use [20, 21]. Recent research using functional imaging has shown that evening chronotypes have altered neural responses to reward compared to morning chronotypes with increased activity in the ventral striatum and decreased reactivity in the medial prefrontal cortex [22] which has previously been associated with increased alcohol consumption [23].

### 1.3. Circadian Misalignment

**Figure 3:**
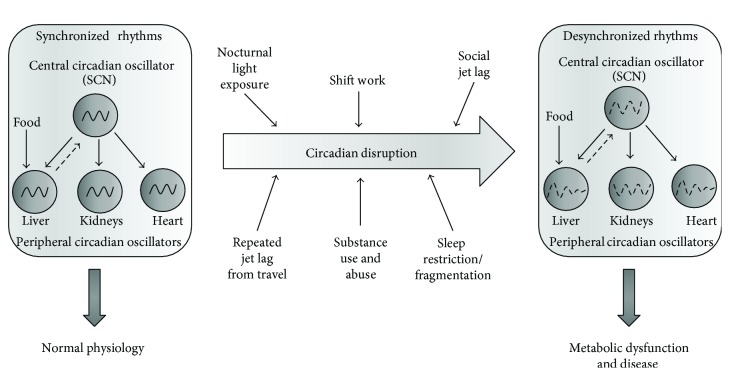
Central and peripheral circadian regulation of metabolic function. Under normal conditions, the central circadian oscillator in the SCN is entrained by light and synchronizes peripheral oscillators. Meal timing can also entrain the liver oscillators. Environmental perturbations such as shift work, jet lag, sleep restriction, and substance abuse create misalignment between the SCN and the peripheral oscillators resulting in metabolic syndromes and disease.

Impairments of the circadian system or desynchronization adversely affects individual health with increased risk of obesity, diabetes, cardiovascular diseases, cancer, and mood disorders [24–28] (Figure 3). Recently, research in animal models and humans has linked circadian dysfunction with increased risk of drug and alcohol abuse. Drug abuse and drug-related pathologies appear higher in populations in which circadian misalignment and sleep deprivation are common [3, 29] including shift workers [30–32] and aging individuals [33]. These substance abuse issues are expected to escalate with increases in the aging population and the proportion of people working extended and rotating shifts [34–36]. Drug abuse and alcohol abuse affect the functioning of the circadian system with the subsequent circadian dysfunction increasing the risks and harms of drug abuse. In this review, we will discuss factors contributing to the increase in sleep and circadian disorders and focus on *Drosophila* as a model for investigating the bidirectional interactions between the circadian clock and drug use.

## 2. Factors Contributing to the Increase in Circadian and Sleep Disorders

### 2.1. Work Schedules

In the past few decades, the number of individuals affected by circadian or sleep disorders has rapidly risen [37]. Insufficient sleep is a pervasive problem affecting approximately 30% of adults and 60% of adolescents [38]. Technological advances and globalization have driven changes in occupational and professional practices with a greater number of individuals working extended hours and shift work. In the United States and other developed countries, approximately 15–30% of the population work irregular or shift work schedules [36, 38] contributing to increased circadian and sleep disorders. Individuals working longer days and extended work weeks have become increasingly more common with more than 18% of the people in the United States working more than 48 hours per week [36]. These problems are compounded by poor entrainment of the circadian clock in modern societies.

### 2.2. Circadian Entrainment and Artificial Light at Night

The circadian system and sleep profiles evolved in coordination with the natural light-dark cycles with light providing the strongest zeitgeber or entrainment signal to the circadian oscillator. With the majority of the world's population now living in urban environments [39], artificial indoor lighting substitutes for the natural light-dark cycle entrainment of the circadian clock. Studies in the United States, Canada, and England found that individuals spend less than 12% of their time outside or less than 1-2 hours per day overall [40–43]. As indoor light levels (100–300 lux) are orders of magnitude lower than those of direct sunlight (10,000 lux range), decreased time outdoors results in weaker signals to the circadian clock and poorer entrainment [44]. In contrast to light signals during the day, light at night shifts the phase of the circadian clock (Figures 1(b) and 1(c)). It has been estimated that 99% of individuals living in the United States and Europe and 80% of the people worldwide experience significant light pollution at night [45, 46]. During the night, light from a full moon is less than 1.0 lux of light, usually 0.1–0.3 lux [45]. However, artificial light at night is considerably higher from a variety of sources including outdoor lighting estimated at 5–15-lux light exposure, indoor evening lighting at 100–200-lux light exposure, and personal electronic use ranging up to 100-lux light exposure [45]. In the past decade, the shift from the use of incandescent light bulbs to the use of fluorescent and LED lights with shorter wavelengths increases the potential for circadian disruption at night as melanopsin, the circadian photopigment, is particularly sensitive to shorter-wavelength blue light [47, 48]. Increased exposure to artificial light at night has been associated with increased risk of cancer, diabetes, obesity, and mood and behavioral disorders [45, 49–51]. Increased artificial light at night [46, 52] combined with reduced individual exposure to daytime sunlight [43] contributes to weakened circadian entrainment and circadian dysfunction. Poor entrainment and low-level circadian function make it more difficult to maintain synchronization of central and peripheral circadian clocks in the face of circadian perturbation.

### 2.3. Evening Chronotype

An individual's chronotype may also increase the risk of circadian desynchronization. Individuals with evening chronotypes are prone to even greater late-night phenotypes with less exposure to sunlight and more reliance on artificial lighting compounding the problem [53]. The phase of the circadian clock changes with development and aging. Whereas young children have a morning chronotype, in teenagers and young adults, the biological clock is naturally shifted by several hours resulting in the prevalence of evening chronotypes in this age group [54–56]. Fixed work and school schedules compound the problem of circadian dysfunction in individuals with an evening chronotype.

### 2.4. Personal Electronics

The use of personal electronics and shifts in activity patterns between weekdays and weekends strongly contribute to the rise in circadian and sleep disorders. The use of smartphones and personal electronics at night has further potentiated circadian disorders and associated problems by increasing exposure to light during the night [45, 57, 58]. Computer and cell phone use at night by adolescents has been correlated with decreased weekday sleep [59]. Teenagers and young adults are particularly susceptible to smartphone dependence [60], with more than 50% of adults and approximately 75% of children and adolescents exhibiting signs of dependence upon their smartphones including anxiety [61]. Smartphone dependence appears almost universal around the world contributing to sleep disorders and poor sleep quality in teenagers, college students, and adults [62, 63].

### 2.5. Social Jet Lag

Social jet lag, defined as a change in activity/rest patterns between workdays and free days, results in individuals continuously undergoing shifts to their circadian clock and a perpetual state of circadian misalignment as peripheral circadian oscillators have insufficient time to resynchronize prior to the next phase shift (Figure 3). Social jet lag is prevalent in adults and adolescents, particularly in individuals with an evening chronotype, with estimates of social jet lag affecting almost 70% of individuals [64]. Social jet lag has been correlated with increased obesity [65], diabetes, cardiac function and heart disease [66, 67], and depression [68]. Higher levels of alcohol use observed in individuals with evening chronotypes may be compounded by social jet lag [18].

## 3. *Drosophila* as a Versatile Model System

### 3.1. Advantages of *Drosophila*

*Drosophila* is an excellent model system for dissecting the bidirectional connections between the circadian clock and drugs of abuse as the signaling pathways that regulate reward processes, addiction, and circadian function are highly conserved between *Drosophila* and mammals [69–71]. The free-running period in *Drosophila* is approximately 24 hours with flies exhibiting crepuscular activity under laboratory conditions including dawn anticipatory activity [72, 73]. The relatively short life cycle, the ability to generate large populations in a short time period, and the low cost of culture and maintenance in *Drosophila* permit complex genetic experiments to be completed in a fraction of the time it would take in vertebrate models [74, 75]. Powerful neurogenetic techniques including forward genetic screens, reverse genetic techniques with genome-wide RNAi lines available, and optogenetic monitoring to assess individual neuronal changes using voltage or calcium sensors have enhanced the utility of the *Drosophila* model [76–81]. *Drosophila* also provides an excellent model to study the complexity of the aging process, offering the ability to characterize single-gene mutations that extend or shorten lifespan [82–85]. Similar to mammals, *Drosophila* shows declines with aging in functional and behavioral performance including sensory functions [86, 87], circadian and sleep-like behavior [88–92], learning and memory [93–95], locomotion [96–98], and organ function [99–101].

### 3.2. Conservation between *Drosophila* and Mammals

The physiological mechanisms underlying most biological processes between *Drosophila* and mammals are remarkably well conserved despite the obvious differences in anatomical structure and complexity [102, 103]. The fly genome contains approximately 14,000 genes, and it is estimated that nearly 75% of the genes implicated in human diseases have functional orthologs in the fly, with 80 to 90% similarity in conserved functional domains at the nucleotide level or protein sequence [104–106]. Anatomically, *Drosophila* has functional equivalents of the mammalian heart [107–109], lung [110, 111], kidney [112, 113], gut [114–116], and reproductive tract [117, 118].

Despite the considerable neuroanatomical differences between flies and mammals, the molecular, cellular, genetic, and electrophysiological properties underlying neuronal behavior and synaptic plasticity also are well conserved [119, 120]. The approximately 100,000 neurons constituting the fly brain form discreet networks that regulate complex behaviors such as sleep [121–123], learning and memory [124, 125], grooming and feeding [69, 126–129], circadian rhythms [130–134], aggression [135, 136], and courtship [137, 138]. The fundamental mechanisms comprising the homeostatic systems and neurochemical circuits are also conserved between *Drosophila* and mammals [69, 139, 140]. At the molecular level, neurotransmission also appears highly conserved from *Drosophila* to mammalian species with classical neurotransmitters including acetylcholine, GABA, glutamate, dopamine, octopamine, serotonin, histamine, and peptide neurotransmitters such as neuropeptide Y/neuropeptide F and insulin-like peptides common to both [69, 141, 142].

The striking mechanistic similarities to mammals have propelled *Drosophila* to the forefront as a competitive model to investigate the link between the circadian system and drug sensitivity, abuse, and addiction. In this review, we highlight *Drosophila* research revealing the interactions between the circadian clock and two drugs of abuse, alcohol and cocaine, and the parallels to mammalian systems.

## 4. *Drosophila* as a Model for the Circadian Clock

Since the 1950s and the pioneering work of Colin Pittendrigh, *Drosophila* has been a prominent model for research defining the conceptual, functional, and molecular basis of the circadian clock [143–147]. The circadian clock in *Drosophila* modulates a broad spectrum of physiological and behavioral processes including locomotor activity, sleep patterns, courtship, learning and memory, feeding behavior, chemosensation, and immune responses [148–157]. As in mammals, the *Drosophila* circadian oscillators also coordinate rhythms in peripheral organs, such as olfactory and gustatory sensitivity rhythms [158–160] and the mitotic response of gut stem cells to damage [161].

Konopka and Benzer isolated the first clock gene mutants in 1971 using forward genetics in *Drosophila* and analysis of the period length of the circadian rhythm in eclosion [162]. Flies with mutations in the gene *period* (*per^L^*, *per^S^*, and *per^01^*) exhibit rhythms in eclosion that are longer, shorter, or arrhythmic, respectively [162]. Identification of *per* spawned additional genetic screens for components of the circadian clock leading to the discovery of *timeless*, *clock*, *cycle*, *doubletime*, *shaggy*, *casein kinase 2* subunits, and *cryptochrome* [163–172]. These studies and subsequent identification of the corresponding genes facilitated research in mammalian systems leading to the discovery of mammalian *per* and *clock* genes, the first circadian genes identified and sequenced in mice [173–175].

**Figure 4:**
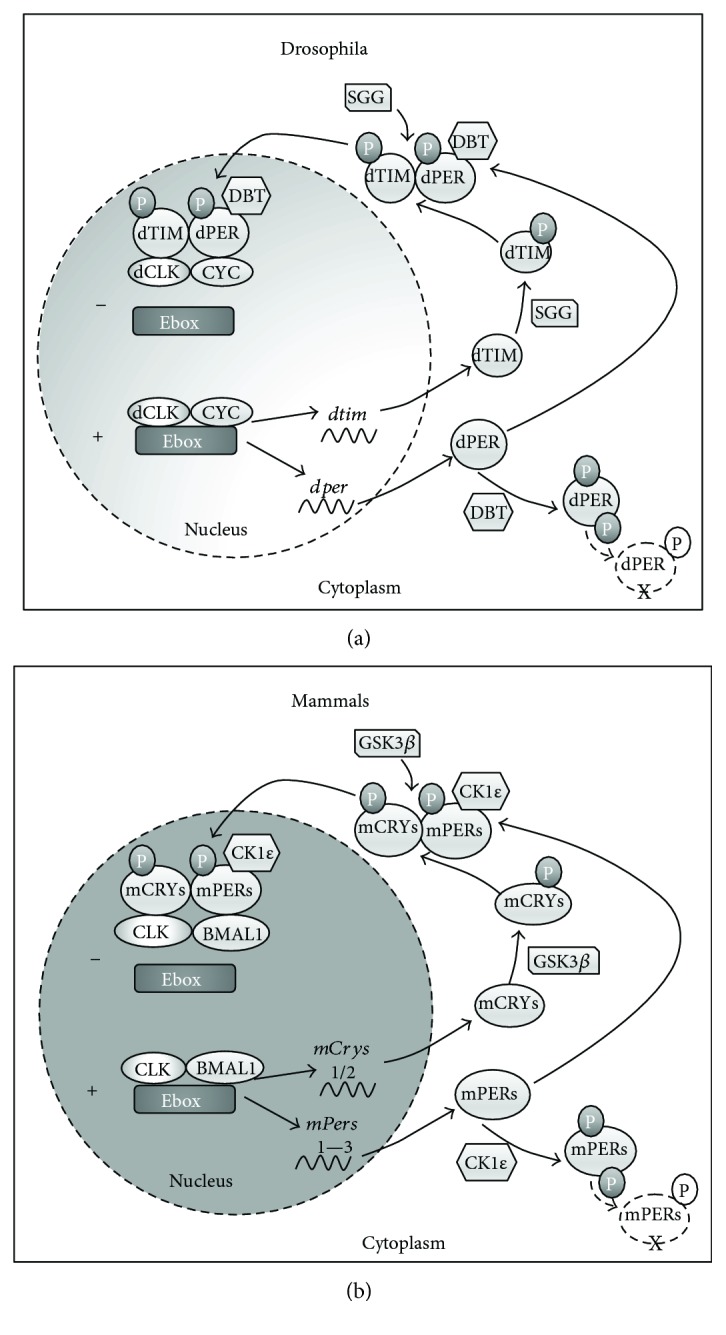
The molecular clocks of *Drosophila* (a) and mammals (b). (a) In *Drosophila*, dCLK and CYC form a dimer, which binds to the E boxes in the promoter of *per* and *tim* as well as to other clock-controlled genes to facilitate transcription. PER and TIM proteins form a complex and translocate into the nucleus providing negative feedback to inhibit dCLK-CYC DNA binding. Phosphorylation mediated by DBT and SGG regulates protein–protein interactions, nuclear translocation, and degradation. (b) In mammals, the transcription factors BMAL1 and CLK form a dimer that binds to E boxes in the promoter of *mPer* and *mCry*. mPER and mCRY proteins form dimers, enter the nucleus, and inhibit the BMAL1-CLK activity. Phosphorylation mediated by CK1 and GSK3 regulates protein–protein interactions, nuclear translocation, and degradation.

The *Drosophila* central brain circadian system comprises approximately 150 clock neurons organized into a network of oscillators: the small and large ventral lateral neurons which control the morning peak of activity and the lateral dorsal and dorsal neurons that control the evening peak of activity [176, 177]. Circadian rhythms generated by both the *Drosophila* and mammalian clock are driven by interlocking autoregulatory transcriptional/translational feedback loops along with posttranscriptional regulatory elements that facilitate the rhythmicity of the clock and generate the 24-hour period [176, 178–183].  Figure 4 provides an overview of the molecular clock in *Drosophila* and mammals. As additional information on the molecular mechanisms of the core circadian oscillators in *Drosophila* and mammals is provided in many excellent reviews [176, 184–186], we only briefly describe the core clock mechanism below.

In *Drosophila*, the positive regulatory elements in the core oscillator are the basic-helix-loop-helix transcriptional elements *clock* (*clk*) and *cycle* (*cyc*) which form a heterodimer and bind to the *per* and *timeless* (*tim*) DNA promoters to activate transcription of the core circadian genes, *per* and *tim* [166, 167, 171], and hundreds of clock-controlled output genes [187, 188]. Monomers of the PER protein are unstable, phosphorylated by *doubletime* (DBT), and targeted for degradation. As dTIM and dPER levels rise, they form a dTIM/dPER/DBT complex which translocates to the nucleus and binds to the dCLK/dCYC complex [165, 172, 189], thereby inhibiting transcription of the *per* and *tim* genes [182, 189]. In mammals, the positive regulatory elements are the transcriptional elements CLOCK (CLK) and BMAL1 (instead of CYC) which form a heterodimer to activate transcription of the clock genes: three orthologs of *period* (m*Per1*, m*Per2*, and m*Per3*) and two *cryptochrome* genes (m*Cry*1 and m*Cry2*) as well as other clock-controlled genes [190, 191]. Following translation of the proteins mPER and mCRY, the proteins dimerize to mediate stability and nuclear translocation and then interact with the mCLK/mBMAL1 complex in the nucleus, inhibiting further transcriptional activation [191]. In flies as in mammals, posttranscriptional elements are necessary for the regulation of protein stability, nuclear entry, and fine-tuning of period length [170, 189, 192]. These include the kinases *doubletime* (DBT), the homolog to mammalian *casein kinase 1 epsilon* (CK1E) [172] which targets dPER for phosphorylation and subsequent degradation [165], and dSHAGGY, a homolog to the mammalian glycogen synthase kinase-3 (mGSK3) [193] which aids in nuclear translocation of the dTIM/dPER/dDBT complex [170, 189, 192]. The above provides a brief outline of the core circadian oscillator with more detailed descriptions of the circadian oscillator and its components in *Drosophila* and mammalian models available in several recent review articles [176, 180, 189, 194].

## 5. *Drosophila* as a Model for Studies of Alcohol Neurobiology

### 5.1. Alcohol-Induced Behaviors

As a model system, *Drosophila* has exemplified the value of invertebrate research and its parallels and conversion into meaningful knowledge in mammalian systems particularly for studies of drugs of abuse [139, 195, 196]. See Table 1 for comparisons of drug-induced behaviors and assays used in *Drosophila* and rodent models. Stereotypical behaviors associated with alcohol exposure are conserved between flies, rodents, and humans [74, 139, 197] including hyperactivity in response to low concentrations of alcohol followed by loss of motor control as alcohol exposure progresses [139, 198–200]. Prolonged exposure to alcohol results in the development of functional tolerance, sedation, and eventually death [139, 198–200]. Similar to mammalian species, *Drosophila* also exhibits sex differences in alcohol sensitivity with males less sensitive to the acute behavioral effects of alcohol but more susceptible to alcohol-induced mortality than females [201, 202].

The molecular and neural mechanisms underlying alcohol-induced behavioral changes appear conserved between flies and mammals [69, 198, 199, 203] making *Drosophila* a practical model for studying the development of functional tolerance, addiction, and reward pathways. Functional tolerance includes the development of rapid and chronic tolerance due to changes in neuronal plasticity rather than changes in the absorbance and metabolism of alcohol [204–206]. Like higher vertebrates, flies develop rapid tolerance following a single alcohol exposure and chronic tolerance with multiple or prolonged alcohol exposure [71, 139, 200]. *Drosophila* demonstrates a preference for alcohol-containing food over non-alcohol-containing food [198, 207–209], although the question has arisen as to whether the underlying preference is due to its caloric value [210]. Recent research has shown that the preference for and voluntary consumption of alcohol in *Drosophila* are experience-dependent based upon previous alcohol exposure [211] and independent of caloric, gustatory, or olfactory biases for alcohol [209]. *Drosophila* also exhibits alcohol addiction-like behavior preferring alcohol-containing food even when accompanied by a noxious stimulus as well as relapse-like behavior with high levels of alcohol consumption after alcohol deprivation [198, 208].

### 5.2. Molecular Pathways in Alcohol Responses

The power of genetic approaches in *Drosophila* has facilitated the identification of molecular and cellular mechanisms that mediate alcohol-induced behavior and neural plasticity [212, 213] with many of the identified genes and molecular pathways playing a similar role in mammalian responses to alcohol [214, 215].

#### 5.2.1. cAMP-PKA Pathway

Mutagenesis studies in flies have provided substantial evidence for the role of the cAMP-protein kinase A pathway in alcohol-induced behavioral responses including the adenylyl cyclase-encoding gene, *rutabaga*; the cAMP phosphodiesterase-encoding gene, *dunce*; and the PKA-C1-encoding gene, *dco* [216]. Increased sensitivity to alcohol-induced sedation is observed in *rutabaga* flies although *dunce* mutant flies exhibit sensitivity to alcohol-induced sedation similar to wild-type flies [216]. Flies with mutations in PKA show altered alcohol sensitivity with mutations in the catalytic subunit (*dco* mutation) making them more sensitive to the sedating effects of alcohol [216] while flies with a mutation in the RII subunit of PKA exhibit reduced sensitivity to alcohol-induced sedation [217]. The preference for alcohol self-administration and potentially the processing of its reward salience are also dependent upon adenylyl cyclase activity [209]. The role of cAMP-PKA signaling in alcohol neurobiology appears conserved across species. Genetic downregulation of the cAMP-PKA pathway in mice through manipulation of G protein transduction increases sensitivity to ethanol while upregulation of adenylyl cyclase activity reduces the sensitivity of mice to alcohol-induced sedation [218]. Mice with a knockout mutation in the RII*β* subunit of PKA exhibit lowered alcohol sensitivity [219] similar to what is observed in flies. The PACAP-like analog *amnesiac* encodes a putative neuropeptide which may trigger the cAMP-PKA pathway via adenylate cyclase activity [220]. Flies with mutations in the *amnesiac* gene also exhibit increased sensitivity to the sedative effects of alcohol [216]. Thus, the cAMP-PKA pathway appears to be a key regulator in behavioral alcohol sensitivity and the preference for alcohol across species.

#### 5.2.2. The LMO-ALK Axis

*Drosophila LIM-domain only* (*dLmo*), also known as Beadex, encodes a transcriptional regulator that affects behavioral responses in the adult fly to alcohol and cocaine. In flies, decreased *dLmo* levels increase alcohol sensitivity with flies sedating more quickly while increased *dLmo* decreases sensitivity to alcohol sedation [221]. Similarly, mice with reduced expression of *Lmo3* exhibit increased sensitivity to the sedating effect of alcohol [221, 222]. *Lmo3*-null mice also drink more alcohol in the “drinking in the dark” test compared to wild-type mice [222]. In a microarray analysis for gene targets with inverse expression of the *Bx* repressor, the *Drosophila* homolog of *anaplastic lymphoma kinase* (*dAlk*) was found to be negatively regulated by *dLmo* [221]. Flies with mutations in dAlk show increased resistance to alcohol-induced sedation [223]. *dAlk* has been shown to modulate *Erk* activity in the fly brain and likely influences sensitivity to alcohol via *Erk* signaling [224]. Similarly, rodent and human orthologs of *dAlk* include *Bx* (*Lmo4*) and *Alk* that have been shown to modulate alcohol sensitivity and consumption and may be involved in alcohol dependence [223, 225–227]. *Alk*-knockout mice also show higher alcohol consumption compared to wild-type mice [223].

#### 5.2.3. GABA Neurotransmission

The effects of alcohol on neurotransmission, particularly on glutamate and GABA neurotransmission, are also conserved across species [69, 71, 215]. Alcohol affects GABA neurotransmission through binding to GABA_A_ and GABA_B_ receptors [215]. In *Drosophila*, GABA_B_ receptor activity mediates alcohol sensitivity and upregulation inhibits rapid tolerance to alcohol exposure [228]. Flies with GABA_B_R1 downregulation through RNAi expression have decreased GABA_B_ receptor function and exhibit decreased alcohol-induced motor impairments [228]. Pharmacological downregulation of GABA_B_ also decreases alcohol sensitivity whereas flies treated with a GABA_B_ agonist (3-APMPA) fail to develop rapid alcohol tolerance [228]. Similarly in mice, the GABA_B_ agonist baclofen blocks the development of rapid tolerance [229] and GABA_B_ antagonists attenuate the acute sensitivity to alcohol [230]. However, the observed effect of baclofen on self-administration of alcohol has varied with some studies indicating that baclofen decreases voluntary alcohol consumption [231, 232] while others demonstrate increased alcohol consumption [233, 234]. This confusion has recently been answered, at least in part, as enantiomer specificity was found to be a critical factor in the directionality of baclofen on alcohol consumption [235]. In alcoholic patients, baclofen has also been shown to reduce alcohol craving [236–240].

#### 5.2.4. Glutamate Signaling

The protein Homer functions as an adaptor protein in the postsynaptic density coupling membrane proteins with downstream signaling, including glutamate receptors. Transcript levels of *homer* decrease in wild-type flies following alcohol exposure, and *homer^R102^* mutant flies demonstrate increased sensitivity and decreased tolerance to alcohol exposure [241]. *Homer2*-KO mice demonstrate reduced voluntary drinking, reduced preference for alcohol, and increased sensitivity to alcohol confirming a conserved role of Homer function in the regulation of alcohol-induced behaviors [242]. More recently, chronic alcohol exposure has been shown to increase *Homer2a/b* and *mGluR1* expression in the nucleus accumbens core (NaCc) and central amygdala (CeA) of rats reinforcing previous research that chronic alcohol induces glutamatergic plasticity in the brain [243, 244].

#### 5.2.5. Potassium Channels

Further evidence for the high degree of conservation in alcohol tolerance arises from the studies of the gene *slowpoke* (*slo*) that encodes the *Big Potassium* (BK) channel-forming subunits. SLO is necessary for the acquisition of rapid tolerance with reduced *slo* expression in flies eliminating the ability to acquire tolerance [245–247]. Increasing *slo* expression in the *Drosophila* brain mimics functional alcohol tolerance [245, 248]. Mammalian BK channels encoded by *slo* are inhibited and potentiated by alcohol [249–251]. Stimulation of BK channels in the rat supraoptic nucleus and striatum increases the response to alcohol-induced tolerance [252]. The actions of alcohol on BK channels are dependent upon the 𝛽1–𝛽4 subunits shown to reduce the potentiation of BK channels following acute alcohol exposure [250, 253–255].

#### 5.2.6. Reward Signaling: Dopamine and NPY

Reward pathways mediating alcohol addiction and abuse are also conserved between flies and mammals [69, 139]. Dopamine is a pleiotropic modulator of behavior strongly implicated in the development of reward and addiction in mammals and flies [256–260]. In flies, dopamine signaling via D_1_ receptors is necessary for alcohol-induced hyperactivity and preference [200, 257, 261] with dopaminergic neurons in the ellipsoid body of the central complex critical for the regulation of alcohol-induced hyperactivity [257]. Similarly, fast and steep increases in dopamine activate low-affinity D_1_ dopamine receptors necessary for the rewarding effects of alcohol and triggering alcohol-induced conditioned responses in mammals [262].

Another neuropeptide, neuropeptide Y (NPY), plays a prominent role in the negative affective behaviors associated with stress and alcohol [263, 264] with a conserved role in reward pathways across species. In mammalian studies, rats selectively bred for high alcohol preferences have low levels of NPY expression in the striatum and increased anxiety-like behaviors [265]. Mice selected for high alcohol preference also show blunted NPY in the nucleus accumbens core and shell in response to acute alcohol exposure compared to control mice [266]. Manipulations of NPF signaling, the *Drosophila* homolog of NPY, affect alcohol preference with inhibition of the NPF pathway enhancing alcohol preference while NPF activation reduces alcohol preference [267]. Flies with loss-of-function mutations in NPF/NPFR1 signaling also exhibit decreased sensitivity to alcohol sedation whereas flies in which NPF is overexpressed show increased sensitivity to alcohol sedation [268].

#### 5.2.7. Cellular Stress Pathways

Genes involved in cellular stress responses may also have a conserved role in the development of alcohol tolerance including heat shock proteins, cytochrome P450 proteins, and glutathione transferases [269]. The *hangover* gene is a Zn-finger transcription factor necessary for cellular oxidative stress responses [270]. Flies with a mutation in *hangover* exhibit decreased rapid alcohol tolerance, although no differences are observed in alcohol-induced sedation [247]. The human ortholog of *hangover* is *ZNF699*, and human studies have identified polymorphisms in this gene associated with alcohol dependence [271, 272]. Another stress-related gene involved in alcohol tolerance is the microtubule-associated protein *jwa* (alias *ARL6IP5*, *addicsin*), which increases in response to oxidative stress and heat shock in mammals [273, 274]. In flies, RNAi-mediated knockdown of *djwa* decreases the development of alcohol tolerance [275]. In mammals, the homologous *addicsin* has been implicated in the development of morphine tolerance [276]. While considerably more research needs to be done, there appears to be a conserved role for proteins involved in cellular stress responses in alcohol and drug tolerance across species.

#### 5.2.8. Regulation of Cytoskeletal Elements

Regulation of actin dynamics also has been implicated in alcohol-induced behavioral responses. The Rho family of GTPases, including Rho1, Rac1, and Cdc42, regulates actin dynamics. In flies, *Ras suppressor1* (*Rsu1*) regulates alcohol sensitivity functioning through the regulation of actin dynamics upstream of Rac1 GTPase [277]. Flies with mutations in *Rsu1* exhibit reduced sensitivity to alcohol and a naïve preference for higher alcohol consumption that remains unchanged with experience [277]. Furthermore, the Rho GTPase activator protein 18B (RhoGAP18B) with three protein isoforms affect actin dynamics of which the RhoGAP18B PC isoform also affects the sensitivity to alcohol-induced sedation and hyperactivity [278, 279]. The loss of the full-length RhoGAP18B PC protein decreases alcohol sensitivity [278, 280]. Human genome-wide association studies have found correlations between *Rsu1* SNP and ventral striatum activity, and a mutation in *Rsu1* is associated with alcohol dependence [277]. Further, activation of the *Arf6* small GTPase results in increased resistance to alcohol-induced sedation, and flies with reduced expression are more sensitive to alcohol [281, 282]. The Efa6 activation of Arf6 is required for normal responses to alcohol-induced sedation as Arf6 and Efa6 mutant flies show reduced sensitivity to alcohol-induced sedation and no rapid tolerance to alcohol exposure [283]. Human genome-wide association studies show correlations between SNPs of Arf6 and Efa6 and increased alcohol drinking behavior [283].

The strikingly similar molecular and cellular mechanisms underlying alcohol-responsive behaviors in flies and mammals validate *Drosophila* as a model for alcohol research. Although obvious neuroanatomical differences exist between mammals and flies, alcohol affects brain regions in flies for which functionally similar parallels can be drawn to specific mammalian brain regions [139, 199]. The brain regions involved in *Drosophila* alcohol neurobiology and detailed description of genes and molecular pathways have been the subject of several recent reviews [71, 139, 199, 200, 284].

## 6. *Drosophila* Links Circadian and Alcohol Neurobiology

The interaction between time of day and the sensitivity to alcohol was described more than a half-century ago with studies in mice detailing time-of-day differences in alcohol toxicity [285]. Since that time, numerous behavioral studies have documented the circadian regulation of alcohol sensitivity and the bidirectional influence of alcohol on the functioning of the circadian clock [286–289]. The emergence of *Drosophila* as a model for alcohol research has expanded the opportunities for defining the bidirectional interactions between alcohol and the circadian clock using behavioral and genetic studies.

### 6.1. Circadian Regulation of Alcohol Behavioral Sensitivity

As the neural circuitry and molecular signaling pathways may differ between alcohol-induced behaviors, the potential for circadian regulation of multiple behaviors has been examined in *Drosophila* including the loss of righting reflex which reflects the loss of postural motor control after alcohol exposure, alcohol-induced sedation, the recovery from sedation, and functional tolerance. In *Drosophila*, the circadian clock differentially regulates acute behavioral sensitivity to alcohol dependent upon time of day with circadian rhythms in alcohol-induced loss of righting reflex and sedation occurring in both light-dark cycles and constant darkness [202, 290]. Flies exhibit the greatest sensitivity to alcohol during the mid-to-late subjective night in correspondence to the flies' inactive phase [202, 290]. The time to recover from the sedative effects of alcohol is also significantly greater at night [202]. Phase-dependent correlation of alcohol sensitivity with activity may be a conserved feature of circadian regulation as mice also exhibit rhythms in alcohol sensitivity with increased sensitivity during the day (inactive period) and decreased sensitivity at night (active period) [291]. Humans show time-of-day rhythms in the consumption of alcohol and alcohol sensitivity [292, 293]. However, not all alcohol-induced behaviors are directly regulated by the circadian clock. In *Drosophila*, the degree of rapid tolerance assessed at four hours does not show dependence upon the time of alcohol exposure, although rhythms are observed in the loss of righting reflex in both the initial alcohol exposure and the test exposure [290]. Alcohol absorbance also does not vary based upon time of exposure [290] or between circadian clock mutants and wild-type flies [294].

### 6.2. Circadian Dysfunction Increases Alcohol Sensitivity

When the circadian clock is rendered nonfunctional through either genetic or environmental manipulations, *Drosophila* exhibits significantly increased behavioral sensitivity to alcohol [202]. *per^01^* mutant flies are more susceptible to alcohol-induced sedation with significantly shorter alcohol exposure required for sedation and longer recovery times to regain postural control compared to wild-type flies [202]. Constant light is frequently used in *Drosophila* as an environmental method to ablate molecular and behavioral circadian rhythmicity without the need for genetic manipulations [150, 290, 295–297] as genetic mutations or constitutive knockouts present during development may affect neural circuitry thus influencing adult behavior. Flies housed in constant light exhibit increased sensitivity to alcohol and longer recovery times [202, 290]. Circadian arrhythmicity arising from the *per^01^* mutation or constant light exposure also appears associated with increased alcohol-induced mortality in *Drosophila* [298].

Disruption of the circadian oscillator affects alcohol behaviors and toxicity across species. *mPer2* mutant mice display increased voluntary alcohol intake and fail to exhibit diurnal rhythms in the behavioral response to alcohol [291, 299]. In humans, the regulation of alcohol consumption appears altered with gene variations in *hper2* determined by single-nucleotide polymorphism analysis correlated with higher or lower alcohol consumption [299, 300]. The Per2 gene and the circadian clock have also been postulated to play a role in regulating developmental changes following alcohol exposure as *mper2* mutant mice fail to exhibit the persistent hypothalamic changes associated with early-life alcohol exposure in wild-type mice [301]. In mice, genetic or environmental disruption of the circadian clock increases alcohol-induced pathologies including intestinal permeability and hepatic inflammation [302] and significantly alters gene expression for many genes involved in inflammation or metabolic responses [303]. Alcohol-induced colorectal cancer in mice is also increased with circadian desynchronization from shifting light-dark cycles [304]. In humans, circadian misalignment in night-shift workers is postulated to be a contributing factor to the development of liver injury with alcohol consumption [30]. Thus, circadian desynchronization appears to be a key factor in alcohol sensitivity and alcohol-induced pathologies across species.

### 6.3. Circadian Clock and Alcohol Tolerance

The development of alcohol tolerance and associated changes in neural plasticity also appears to require a functional circadian clock, even though the degree of rapid tolerance following alcohol exposure does not vary with time of day [290]. Flies with mutations in core oscillator genes including *per^01^* and *tim^01^* fail to develop functional tolerance following alcohol exposure while *cyc^01^* flies only acquire weak tolerance [294]. Flies with the circadian clock rendered nonfunctional by constant light also fail to develop tolerance [294]. However, mutant *Clk^Jrk^* flies develop tolerance similar to that of wild-type flies raising the possibility that PER and other core clock components regulate alcohol-induced behaviors independent of the circadian clock [294].

### 6.4. Effect of Alcohol on the Circadian Clock

The interactions between the circadian clock and alcohol are bidirectional. In rodent models, acute and chronic alcohol exposure results in phase shifts in locomotor activity rhythms and alters the ability of the circadian system to respond to perturbations [305–308]. Furthermore, chronic alcohol administration alters SCN function by disrupting the SCN responsiveness to light and nonphotic resetting as shown through *in vivo* and *in vitro* studies [305, 309]. Chronic alcohol exposure also affects neuropeptide signaling in the SCN decreasing the amount of vasopressin and VIP in SCN neurons [286]. In humans, chronic alcohol use or acute binge alcohol consumption appear to be strongly linked to sleep and circadian disorders [3, 310, 311]. The mRNA expression of *clk1*, *bmal1*, *per2*, *cry1*, and *cry2* is significantly reduced in alcoholic patients [312]. However, unlike rodent models, a single acute alcohol exposure in humans does not appear sufficient to affect the phase-shifting ability of the circadian system [313].

Alcohol differentially affects central and peripheral oscillators. Studies in mice and rats demonstrate that repeated alcohol exposure results in major alterations in peripheral rhythms reducing the correlation in the phase relationships between body temperature and activity rhythms as well as altering and blunting the rhythms in plasma corticosterone, glucose, lactic acid, triglycerides, and cholesterol [287, 314, 315]. Distinct tissue-specific interactions and changes in gene expression have also been observed following chronic alcohol use in *clock* mutant mice for the hippocampus, liver, and colon [303]. Alcohol administration increases CLOCK and PER2 protein levels in intestinal epithelial cell cultures and increases measures of alcohol-induced permeability [316]. At the molecular level, chronic alcohol exposure appears to have the greatest effect on phase shifts or disruptions of core clock and clock-controlled genes in the liver rather than in the SCN [315, 317]. Chronic alcohol phase advances the rhythms in *Per1* expression in the adrenal and pituitary clocks and *Per2* expression in the liver clocks, without affecting the molecular clock in the SCN resulting in discord in the phase relationships between the SCN and peripheral oscillators [314, 317]. However, earlier research suggested that chronic alcohol administration affected the rhythmic expression of proopiomelanocortin, Per2, and Per3 in the SCN [318].

Bidirectional interactions between alcohol and the circadian clock are also conserved in *Drosophila*. For example, developmental alcohol exposure during the third larval instar affects period length in adult flies [319, 320]. With substantial evidence attesting to the value of *Drosophila* as a model for alcohol research and the parallels between the interactions of the circadian system with alcohol neurobiology across species (see Table 2), *Drosophila* appears poised for future studies probing the mechanism through which the circadian clock modulates these behaviors.

## 7. Interactions of the Circadian System with Cocaine

### 7.1. Circadian Modulation of Cocaine Behaviors

One of the earliest hints of an interaction between cocaine and the circadian system arose from studies of patients with seasonal affective disorder and seasonal variations in cocaine abuse [321, 322]. Subsequent research in rodent models found that the effects of acute cocaine exposure on locomotor activity were dependent upon the time of day [323] as was cocaine-induced behavioral sensitization [324–326]. Similar to alcohol behaviors, cocaine sensitization appears lowest during the night [324–326] correlated with the animal's activity period. Cocaine self-administration also varies with the circadian cycle [327] suggesting strong interactions between cocaine and the circadian system.

### 7.2. Cocaine's Effects on the Circadian Clock

Similar to alcohol, cocaine also bidirectionally interacts with the circadian clock. Cocaine administration disrupts light-induced phase shifts of the SCN during the night while cocaine administration during the day induces phase advances as shown through *in vivo* studies in mice and *in vitro* studies using SCN slices [328, 329]. The effects of cocaine on phase shifting of the circadian clock appear mediated through serotonin transporter antagonism and Per2 [329]. Repeated cocaine exposure has also been shown to differentially affect *per2* gene expression in the caudate putamen in rats [330].

### 7.3. Genes Mediating Circadian Cocaine Responses

At the first glance, *Drosophila* may seem an unusual model to forward the studies of drug abuse, but the extent of readily available mutants and the ease of behavioral screens accelerated the identification of genes involved in circadian-cocaine interactions (Table 3). In response to cocaine, *Drosophila* exhibits motor and reflexive behaviors including grooming and locomotor circling similar to those of mammals [331–333]. One of the first studies demonstrating the interplay between circadian genes and the drugs of abuse showed that flies with mutations in the circadian gene *per* failed to sensitize to cocaine (indicated by erratic jumping, twirling, and paralysis) even after repeated exposures to cocaine compared to wild-type Canton-S flies [334]. However, *per* mutants with altered period length rather than arrhythmicity display differential responses to cocaine; *per^S^* mutants exhibit increased responsiveness followed by a weak sensitization to cocaine exposure while *per^L^* mutants show normal initial behavioral responses to cocaine but no sensitization [334].

This research spurred subsequent studies in mice examining the relationship between *per* and cocaine responsiveness. In mice, the *period* gene also appears strongly linked to cocaine behaviors. Mice with a mutation in *mPer1* do not sensitize to cocaine [324], and circadian rhythms in Per1 expression in the striatum appear necessary for rhythms in cocaine sensitization [326]. Recently, a variable repeat polymorphism in hPer2 was correlated with higher expression in cocaine-addicted individuals and cocaine users [335]. *mPer2* mutant mice display hypersensitization to cocaine, although they exhibit normal levels of conditioned place preference (CPP) with cocaine reward [324].

Additional evidence for the role of PER in cocaine responses comes from studies of flies with mutations in proteins that interact with PER. Flies with a mutation in *doubletime (homolog of casein kinase 1-epsilon)* require a higher dosage of cocaine to exhibit cocaine-induced behaviors with the first cocaine exposure but do not show significant sensitization with multiple exposures [334]. CLK and CYC mutant flies display increased initial sensitivity to cocaine compared to wild-type flies but fail to develop sensitization following the second exposure [334]. In mammals, additional circadian genes have also been implicated in cocaine responses. *Clock^Δ19^* mutant mice exhibit increased cocaine self-administration and conditioned place preference in response to cocaine compared to wild-type mice [336, 337].

While the above studies highlight the role of multiple circadian genes, particularly *per*, in mediating drug-induced behaviors, the function of these genes in drug behaviors may be distinct from their function in the regulation of the circadian clock. For example, *tim^0^* flies exhibit behavioral responses to cocaine similar to wild-type flies suggesting a divergence between the regulation of circadian function and that of cocaine behavior [334]. Intriguingly, regulation of cocaine behaviors appears to involve the small ventral lateral neurons in *Drosophila*, considered circadian pacemaker neurons, although neurotransmission through the primary circadian neuropeptide PDF is not required for cocaine behavioral responses [333]. Within the small ventral lateral neurons, the Lim-only gene *lmo* appears involved in both cocaine sensitization and circadian locomotor activity rhythms. *lmo* expression/function is inversely correlated with cocaine sensitivity as mutants with low levels of LMO exhibit increased cocaine sensitivity while flies in which overexpression of LMO occurs demonstrate increased resistance to the acute effects of cocaine [333]. *lmo* mutant flies display poor locomotor activity rhythms. In mice, *lmo4* also regulates cocaine sensitization [221] and the expression of *lmo4* is regulated by the circadian clock [338] reinforcing the relationship between the circadian clock and cocaine behaviors.

### 7.4. Circadian Regulation of the Reward System

A conserved feature of circadian influence on drug abuse and addiction arises from circadian regulation of the reward system. Biogenic amines produced in both the central and peripheral nervous system control motor behaviors in vertebrates and invertebrates [339–342]. Cocaine and other drugs of abuse act directly on the mesolimbic dopamine system and other pathways to promote drug-seeking behavior. In mammals and flies during reward learning, the valence and reward properties of a stimulus involve dopamine signaling, glutamate, and GABA in a complex feedback and feedforward network [343, 344]. In *Drosophila*, reward learning requires dopaminergic projections to the mushroom body neurons [125, 345] whereas in mammals, dopaminergic innervation from the ventral tegmental area to the striatum, the bed nucleus of the stria terminalis, and the nucleus accumbens is required for reward preference [346].

In *Drosophila* and mammals, the responsiveness of dopamine receptors is regulated by the circadian clock and dependent on functional expression of the *per* gene [347–349]. Using direct application of a D2 agonist, quinpirole, to the D2 receptors of the ventral nerve cord, Andretic and colleagues found that functional circadian genes are necessary for behavioral responses to cocaine in behaviorally active decapitated flies [334]. Furthermore, flies with mutations in *per*, *clock*, or *cycle* show no induction of tyrosine decarboxylase activity (TDC) which is necessary for the synthesis of tyramine, an important element for cocaine sensitization in *Drosophila* [332, 334]. Similarly in mammals, mice mutant in *Clock* show increased tyrosine hydroxylase (TH), the rate-limiting enzyme for dopamine synthesis as well as other dopamine-related genes, and increased cocaine CPP [336]. Furthermore, many of the diurnal differences in cocaine self-administration may be due to the regulation of dopaminergic transmission. Andretic and Hirsh [347] identified diurnal regulation of dopamine receptor responsiveness in *Drosophila*. Likewise in mammals, most of the components of dopaminergic transmission including the dopamine receptor, dopamine transporter, and tyrosine hydroxylase exhibit diurnal rhythms [350–352]. More detailed discussions of the interactions of the circadian system with drug neurobiology in mammals may be found in recent reviews [348, 353–355].

*Per1*-knockout mice fail to display conditioned place preference (CPP) with cocaine reward [324] which is regulated by the circadian clock through the pineal gland and melatonin [356]. Recently, melatonin also was shown to significantly reduce motivation for cocaine and cocaine-seeking behavior in rats [357]. In summary, these studies suggest that a functional core circadian oscillator is necessary to drive pineal gland/melatonin outputs that regulate striatal *per1* gene expression to affect cocaine behaviors. However, the relationship between *per1* regulation and cocaine behavior is complicated as *Per1* mutant mice self-administer cocaine and display reinstatement of cocaine administration following extinction similar to wild-type counterparts [358]. This is reminiscent of the differential circadian modulation of alcohol-induced behaviors and suggests different neurobiological mechanisms underlying various drug behaviors.

### 7.5. Impact of *Drosophila* Circadian Cocaine Research

Despite the successes with the use of *Drosophila* as a model for investigations of drug abuse, surprisingly little research in *Drosophila* has been performed in the past few years delineating additional circadian-drug interactions outside of alcohol neurobiology. However, the research in *Drosophila* identifying links between PER and cocaine sensitization directly fostered research in mammals investigating circadian interactions with morphine [359–361] and methamphetamine [362]. Thus, research in *Drosophila* has provided impetus and conceptual advances in our understanding of the influence of the circadian clock on behavioral responses to drugs as well outlining roles circadian genes and neurons can play outside of the circadian clock in drug responses.

## 8. Potential Avenues for Future *Drosophila* Research

Despite the progress in research outlining connections between the circadian system, substance abuse, and the reward system, our understanding of the scope of these interactions and the underlying mechanisms through which these connections occur remains limited in both *Drosophila* and mammalian models directly impacting the prevention and treatment of drug-induced pathologies and addiction disorders. Techniques in rodent models have rapidly advanced over the past decade with sophisticated innovations permitting tissue-specific manipulations in gene expression and neuronal activity. Despite these advances, research in rodent models remains expensive and time consuming, reinforcing a need for the continued use of alternative model systems. The ease of maintenance, relatively short lifespan, and the neurogenetic approaches possibly have permitted *Drosophila* to remain at the forefront of neuroscience and disease research facilitating more targeted research in mammalian models. Primary areas of circadian-drug research in which *Drosophila* could provide advancement can be grouped into three strategic classifications: (1) system-level research (behavioral sensitivity and pathology) for defining the interactions between the circadian clock, sleep, and substance abuse; (2) identification of molecular networks for identifying the connections between the circadian system and substance neurobiology; and (3) drug discovery and small-molecule screening for therapy development.

### 8.1. System-Level Research

Behavioral research has exposed bidirectional interactions between the circadian system and substance abuse; however, the scope of these interactions remains undefined. Alcohol and cocaine represent the only drugs of abuse for which circadian interactions have been studied in *Drosophila*. Yet, considerable research has been done on dopaminergic signaling and reward pathways in *Drosophila* with significant parallels shown to mammals making *Drosophila* a suitable choice for studies of additional drugs of abuse. For example, dopamine and octopamine modulate the acute activating effects of nicotine on locomotion and the startle response [261, 363]. As substance abuse is often comorbid with additional substance abuse, poor nutrition, or sleep disorders, the ease of large-scale behavioral studies in *Drosophila* facilitates combinatorial studies.

The effects of other drugs of abuse including nicotine, morphine, amphetamine, and cannabinoids have been studied in *Drosophila* [364–366], expanding the possibilities for further investigation of the bidirectional interactions between the circadian clock and drug neurobiology using *Drosophila*. Comparatively little research has been done to dissect the relationship between endocannabinoid or cannabinoid use and circadian clock function in humans or rodent models [367–369]. However, research has shown that cannabinoids can excite circadian clock neurons, and this may be linked to the behavioral effects of time dissociation experienced by marijuana users [370, 371]. Given the increasing prevalence of marijuana use with the number of users in the United States more than doubling since 2002 to 9.5% of the adult population and approximately 30% of those individuals meeting the criteria for addiction [372], more research is needed on the interactions of marijuana with the circadian clock. The physiological activities of endocannabinoids on cell signaling appear conserved between *Drosophila* and mammalian systems [373], and *Drosophila* has been used to investigate the role of cannabinoids as therapeutics [374, 375]. As concurrent use of marijuana and alcohol increases the effects of the individual drugs [376–378], combinatorial studies are needed. Thus, *Drosophila* may be a practical model for fast high-throughput studies translatable to mammalian models.

Considerably more behavioral research is needed to identify circadian modulation of sensitivity or toxicity encompassing multiple exposure paradigms across age groups. The circadian system weakens with age across species resulting in damped molecular rhythms and altered behavioral and metabolic rhythms [89, 379, 380]. In both humans and animal models, older subjects demonstrate greater difficulty in phase shifting after perturbations to the circadian system [381, 382]. The weakening of the circadian system with age may contribute to the increased sensitivity to or toxicity of drugs of abuse observed in older individuals. In rodent models, aged animals appear more sensitive to the effects of alcohol and alcohol withdrawal [383, 384], although little research has been done examining circadian interactions with alcohol or drugs of abuse in aged animals. With its relatively short lifespan, *Drosophila* is an excellent system for the aging system with analogous age-related changes to those observed in rodent models and humans.

### 8.2. Molecular Networks

With approximately 20,000 estimated human genes and an untold number of regulatory elements [385], identifying the underlying molecular or genetic mechanisms for complex behavioral and physiological issues remains an enormous challenge without potential candidates identified from animal models. This is particularly true for substance abuse disorders affecting the central nervous system that also result in widespread damage across tissues. Despite the neuroanatomical and morphological differences separating flies from humans, parallels exist for disease research affecting the central nervous system, heart, liver, kidneys, and gut [386], crucial organs for understanding the addictive and pathophysiological impacts of drug abuse.

*Drosophila* orthologs have been identified for approximately 75% of known human disease genes [105, 386–390]. The rapid cross-species translational value of *Drosophila* research has been demonstrated in alcohol neurobiology through the identification of the epidermal growth factor signaling pathway [195] and the role of a tyrosine kinase receptor, anaplastic lymphoma kinase [223], and the transcriptional regulator *Lmo* in alcohol behaviors [221]. Likewise, *Drosophila* has provided a model for the identification of candidate genes involved in addiction and reward behaviors [71, 139, 391]. The wide repertoire of tools in *Drosophila* to permit cost-effective large-scale genetic screens includes genome-wide RNAi screens with available collections of RNAi transgenic lines against every *Drosophila* gene [392], complete sets of micro RNA sponges with conditional expression possible [124, 393], and CRISPR-mediated mutations [394, 395].

### 8.3. Drug Discovery

The identification of new drugs for pharmaceutical use starting with target identification or small-molecule screening is a lengthy and expensive process often lasting more than a decade with costs up to $1 billion [396]. To streamline this process, high-throughput screens in *Drosophila* and other invertebrate models such as *C. elegans* have been employed more frequently in the past few years as a platform for target identification, drug discovery, and small-molecule screening. Previous research has demonstrated the predictive validity of *Drosophila* in preclinical research. *Drosophila* has proven beneficial for the validation and development of cancer drugs as well as for screening previously approved drugs for alternate purposes [397]. The tractability of *Drosophila* for large-scale screens include (1) viability and development assays for embryos, larvae, pupae, and adults; (2) whole-organism drug screens for absorption, metabolism, or toxicity; and (3) reporter assays including luciferase or GFP expression assays [397]. With the high degree of phylogenetic conservation in cellular signaling pathways, mechanistically the similarities between the *Drosophila* and mammalian circadian system make *Drosophila* an ideal platform for drug discovery for the identification of potential targets or therapeutics impacting the circadian system.

Through the ages, technological innovations have engineered societal changes transforming cultural norms and causing the urbanization of societies. Rapid advances in communication, networking, and information dissemination in the past two decades have solidified the establishment of a 24/7 global society further contributing to the rise of individual circadian and sleep disorders. The swiftness with which these technology-driven societal and cultural changes have become entrenched in children, adolescents, and adults makes it unlikely that the physical and mental health problems arising from circadian and sleep disorders will vanish. Thus, there is a critical need for continued research to delineate the mechanisms through which the circadian clock or circadian dysfunction affects substance abuse and conversely how substance abuse contributes to alterations in the functioning of the circadian system. Renewed research emphasis on invertebrate models as a practical and economical model to tackle these problems will provide basic biological insights into molecular pathways and cellular interactions associated with defined behaviors that can subsequently be investigated in more complex model systems with rapid translational impacts. Research in *Drosophila* has the capability to advance the understanding of the molecular changes or the genetic risk factors that transform substance use to abuse and addiction potentially providing new avenues for the identification of therapeutic interventions to minimize the risk of drug abuse and drug toxicity.

## Figures and Tables

**Table 1:** Behavioral measures and assays of drug addiction in *Drosophila* and rodents.

Behavior	Assay	Examples of research studies	
		*Drosophila*	*Rodents*
*Reward/preference* Induced state that leads to conditioned reinforced behavior	(1) Self-administration (2) Electrical stimulation (3) Conditioned place preference (4) Conditioned taste preference (5) Conditioned taste avoidance	(1) [198, 207, 211] (2) [200] (3) [198, 200] (4) [198, 398] (5) [198]	(1) [399] (2) [400, 401] (3) [402, 403] (4) [399, 404] (5) [405, 406]
*Drug seeking* Affective state inferred from increased behavioral responses to drugs, drug-associated cues, or stress	(1) Self-administration (2) Electrical stimulation	(1) [198] (2) [200]	(1) [407, 408] (2) [409, 410]
*Functional tolerance* Adaptations in neural function *Rapid tolerance* Following a single acute exposure when drug has metabolized *Chronic tolerance* Following prolonged or repeated drug exposures	(1) Injection behavioral assays (2) Self-administration (3) Sedation and negative geotaxis assay	(1) [228] (2) — (3) [204, 205, 280]	(1) [405, 411] (2) [412] (3) [413, 414]
*Sensitization* Increased motor-stimulant response following repeated drug exposures	(1) Locomotor activity test	(1) [332, 334, 415, 416]	(1) [324, 417–420]
*Withdrawal* Aversive state that motivates drug seeking	(1) Conditioned place aversion (2) Sedation and negative geotaxis (3) Self-administration	(1) — (2) [421, 422] (3) —	(1) [423] (2) [424, 425] (3) [426, 427]
*Relapse/reinstatement* Spontaneous recovery of drug seeking after abstinence or extinction of behavior may be triggered by cues previously paired with drug use or stress	(1) Self-administration (2) Electrical stimulation (3) Injections	(1) [198, 428] (2) — (3) —	(1) [429, 430] (2) [431, 432] (3) [433]

**Table 2:** Genes that mediate circadian and alcohol interactions.

Fly ortholog	Encoded protein	Genetic manipulation	*Drosophila* alcohol-related phenotypes	Mammalian homolog	References
*per^01^*	PER	↓ expression	↑ alcohol sensitivity ↓ rapid tolerance ↑ recovery time	mPer1; mPer2	[202, 290, 291, 294, 299, 301, 318]
*tim^01^*	TIM	↓ expression	↑ alcohol sensitivity ↓ rapid tolerance	—	[294]
*cyc^01^*	CYCLE	↓ expression	↑ alcohol sensitivity ↓ rapid tolerance	BMAL	[30, 294]
*Clk^JRK^*	CLOCK	↓ expression	No change	CLOCK	[294, 434]

**Table 3:** Genes mediating circadian and drug interactions in flies and mammals.

Gene/manipulation	Mechanism of action	Drug-related phenotypes in *Drosophila*	Reference	Drug studied in mammals	Reference
*per^01^*	Regulation of circadian rhythms	↓ behavioral sensitization to cocaine	[334]	*mPer1* and *mPer2*: cocaine, morphine, and amphetamines	[324, 326, 336, 435, 436]
*clk*	Regulation of circadian rhythms	↓ behavioral sensitization to cocaine	[334]	*Clk*: cocaine, morphine, and amphetamines	[324, 336, 437, 438]
*cyc^01^*	Regulation of circadian rhythms	↓ behavioral sensitization to cocaine	[334]	*Bmal*: cocaine and amphetamine	[324, 336, 436]
*tim^01^*	Regulation of circadian rhythms	No change in response to cocaine	[334]	—	—
*dbt*	Regulation of circadian rhythms	↓ behavioral sensitization to cocaine	[334]	*Csnk1*𝜀: cocaine, amphetamines, and opiods	[439–442]
*dLmo*	Regulation of dopamine receptor expression	↑ sensitivity to cocaine and nicotine and weak circadian rhythms in locomotor activity	[333]	*Lmo4*: cocaine	[338, 443]
